# Comparison of Bioelectrical Impedance Vector Analysis (BIVA) to 7-point Subjective Global Assessment for the diagnosis of malnutrition

**DOI:** 10.1590/2175-8239-JBN-2021-0099

**Published:** 2021-09-24

**Authors:** Clara S. A. Sugizaki, Nayara P. Queiroz, Débora M. Silva, Ana T. V. S. Freitas, Nara A. Costa, Maria R. G. Peixoto

**Affiliations:** 1 Universidade Federal de Goiás, Faculdade de Nutrição, Programa de Pós-graduação Nutrição e Saúde, Goiânia, GO, Brasil.; 2 Universidade Federal de Goiás, Faculdade de Nutrição, Goiânia, GO, Brasil.

**Keywords:** Renal Insufficiency, Chronic, Malnutrition, Nutritional Assessment, Renal Dialysis, Insuficiência Renal Crônica, Desnutrição, Avaliação Nutricional, Diálise Renal

## Abstract

**Introduction:**

Bioelectrical impedance vector analysis (BIVA) is a non-invasive and low-cost strategy. The methods used to assess malnutrition in patients undergoing HD are still a challenge. The aim of the present study was to compare BIVA to 7-Point Subjective Global Assessment (7-point SGA) to identify malnutrition. We also investigated the sensitivity and specificity of the previously proposed cutoffs point for BIVA parameters.

**Methods:**

Patients of both sexes, over 20 years of age, on HD treatment were included. Anthropometric parameters, laboratory data, and bioelectrical impedance analysis (BIA) were evaluated. Values of resistance (R) and reactance (Xc) obtained by mono-frequency BIA were normalized to body height (H) to generate a graph of the bioimpedance vector with the BIVA software. The analysis of the area under the receiver operating curve ROC (AUC) was performed.

**Results:**

Among the included 104 patients, the mean age was 51.70 (±15.10) years, and 52% were male. The BIVA had a sensitivity of 35% for diagnosing malnutrition. The specificity of BIVA for identifying the well-nourished patients was 85.7%. The diagnostic accuracy between the BIVA and 7-point SGA was AUC=0.604; 95%CI 0.490-0.726, higher than the previously established cutoff values (AUC=0.514; 95%CI: 0.369-0.631). The 95% confidence ellipses did not overlap (p<0.05).

**Conclusion:**

Our study showed low accuracy of BIVA for diagnosing malnutrition using a 7-point SGA as a reference standard. However, it is a complementary method for assessing nutritional status as it provides data on cellularity and hydration, which are important aspects for the HD population.

## Resumo

**Introdução:**

Análise vetorial de impedância bioelétrica (BIVA) é uma estratégia não invasiva e de baixo custo. Os métodos usados para avaliar desnutrição em pacientes em HD ainda são um desafio. O objetivo do presente estudo foi comparar BIVA com Avaliação Subjetiva Global de 7 pontos (ASG de 7 pontos) para identificar desnutrição. Também investigamos sensibilidade e especificidade do ponto de corte proposto anteriormente para parâmetros de BIVA.

**Métodos:**

Foram incluídos pacientes de ambos os sexos, acima de 20 anos, em HD. Foram avaliados parâmetros antropométricos, dados laboratoriais e análise de impedância bioelétrica (BIA). Valores de resistência (R) e reatância (Xc) obtidos por BIA de mono-frequência foram normalizados para altura corporal (H) gerando um gráfico do vetor de bioimpedância com a ajuda do software BIVA. Foi realizada uma análise da área sob a curva ROC (AUC).

**Resultados:**

Entre 104 pacientes incluídos, a idade média foi 51,70 (±15,10) anos, e 52% eram homens. BIVA demonstrou sensibilidade de 35% para diagnosticar desnutrição. A especificidade da BIVA para identificar pacientes bem nutridos foi 85,7%. A precisão diagnóstica entre BIVA e ASG de 7 pontos foi AUC=0,604; IC95%: 0,490-0,726, superior aos valores de corte estabelecidos anteriormente (AUC=0,514; IC95%: 0,369-0,631). Elipses de confiança de 95% não se sobrepuseram (p<0,05).

**Conclusão:**

Nosso estudo mostrou baixa precisão da BIVA para diagnóstico de desnutrição usando ASG-7 pontos como padrão de referência. Entretanto, é um método complementar para avaliar estado nutricional, pois fornece dados sobre celularidade e hidratação, aspectos importantes para a população em HD.

## Introduction

Malnutrition results from the decrease in ingestion or absorption of nutrients that lead to changes in body composition and cellularity, with a consequent decrease in functional capacity^1^. Patients on hemodialysis (HD) have increased risk for malnutrition due to the disease and treatment characteristics, such as acidemia, altered responses to anabolic hormones, increased levels of non-excreted toxins, blood loss and loss of nutrients in the dialysate^2^. The prevalence of malnutrition in HD patients can reach 50%^3^ and is malnutrition with low quality of life, comorbidities, and mortality^4^.

The criteria or methods used in the screening, assessment, and diagnosis of malnutrition in this group remains a challenge^4^. The most used validated methods are the malnutrition and inflammation score (MIS)^4^, the criteria of the International Society of Renal Nutrition and Metabolism (ISRNM)^5^, and the 7-Point Subjective Global Assessment (7-point SGA)^4^. One of the most suitable criteria for HD patients is the 7-point SGA^4^. Despite its limitations related to its qualitative character, in which the diagnostic accuracy depends on the experience of the observer^6^, the assessment provided by the 7-point SGA was predictive of all-cause mortality in HD patients two years after the initial assessment, adjusted for significant confounding factors^7^. In addition, the 7-point SGA was recently highlighted in the new KDOQI nutritional assessment guidelines, which recommend the tool for nutritional status assessment with a level of evidence of 1B^4^.

On the other hand, the hydration status of individuals on HD can complicate nutritional diagnosis due to hypervolemia, which can mask some anthropometric parameters and, therefore, affect their interpretation^8^. These patients often experience changes in body fluids due to inadequate sodium and fluid excretion and often have decreased body cell mass (BCM) and expansion of the extracellular space^8^.

In this regard, the bioelectrical impedance vector analysis (BIVA) is a non-invasive and low-cost strategy for evaluating HD patients^9^. It is a technique for semi-quantitative assessment of cellularity and body hydration, using bioelectrical impedance analysis (BIA) measurements, such as resistance (R), reactance (Xc) or impedance (Z), which are first normalized by body height (H) and then plotted on a graph of concentric tolerance ellipses indicating nutritional status and hydration^9^. The R vector measures the opposition to the flow of electric current through the intra and extracellular media of the body, is inversely associated with the hydration level of these media^10^. The Xc vector measures the opposition to the current flow caused by the capacitance produced by the membrane cell^10^. Cellularity refers to cells that influence the metabolism in muscles, internal organs, and the nervous system. The BCM is relevant because it measures the metabolically active mass^11^, and malnutrition is associated with a decrease in BCM^12^. BIVA also provides the change in hydration after an HD session^13^.

The available scientific evidence supports the use of BIA for the assessment of body composition in accordance with the KDOQI^4^. Despite the promising characteristics of BIVA for use in the HD population, previous studies analyzing the potential of BIVA for malnutrition diagnosis using SGA as a reference standard are scarce and inconclusive. Piccoli et al.^13^ identified a moderate association on 130 patients (94 males) in HD. Silva et al.^15^ concluded that BIVA parameters demonstrated low to moderate accuracy in men (n=60) and low accuracy in women (n=41) for malnutrition diagnosis. These authors also established BIVA cutoff points for determining malnutrition^14^. Therefore, in the present study, we investigated the BIVA for diagnosing malnutrition compared with the 7-point SGA in HD patients. We also investigated the sensitivity and specificity of the proposed cutoff points for BIVA parameters^14^.

## Methods

### Study design and participants

A cross-sectional study with a convenience sample recruited from two HD centers was conducted. Data collection occurred in 2015. Patients of both sexes, in HD for at least 3 months, and over 20 years of age were included. The exclusion criteria were recent hospitalization (<3 months), use of a pacemaker, presence of infectious diseases, hepatitis, or cancer, and physical disabilities, cognitive impairment, or refusal to participate. The protocol was approved by the Ethics Committee of our institution (54523116500005083).

### Clinical, anthropometry, and laboratory data

Clinical data, the etiology of chronic kidney disease (CKD), the presence of comorbidities, biochemical parameters (hemoglobin, albumin, creatinine, and urea) and KT/v were taken from the patients' medical records. Hemoglobin was determined by the electronic counting method, serum albumin by the enzymatic colorimetric method, creatinine by optical microscopy, and urea by UV kinetics. Anthropometric measurements were taken after the second HD session of the week^15^. Weight and height were collected according to standardized procedures^16^.

### 7-Point subjective global assessment

The 7-point SGA^17^^,^ 18 was performed on the same day as the anthropometric measurements and the following parameters were evaluated: weight change, dietary intake, gastrointestinal symptoms, functional capacity, diseases or comorbidities affecting nutritional needs, and physical examination. To each condition was assigned a score ranging from 1 to 7^6^^,^ 19. In the analysis, patients were categorized as well-nourished (6 and 7 points) and malnourished (1 to 5 points)^14^.

### Bioelectrical impedance analysis (bia) and bioelectrical impedance vector analysis (biva)

The body composition (lean body mass, fat mass, total body water and phase angle) of patients was obtained using tetrapolar mono-frequency bioimpedance (Quantum II - RJL Systems^((r))^, CA, USA, 50 kHz, 800 µA). The electrodes were placed on the midline between the protruding ends of the radius and ulna of the wrist and midline between the medial and lateral malleoli of the ankle on the side of the body without vascular access. Each patient underwent bioelectrical impedance measurements at the beginning of and after the intermediate HD session followed by 20 min of rest, according to a previously published method^20^. R and Xc were used to estimate phase angle $\mathrm{PA}\left(\circ \right)=\arctan\mathrm{gent}\;[(\mathrm{Xc}(\Omega )/R(\Omega ))\times \;(180/\varpi )]$ 21. Z was obtained by the equation: $Z=\sqrt{\left(R^{2}+{\mathrm{Xc}}^{2}\right)}$ . R, Xc, and Z were standardized by height in meters: R/H, Xc/H, Z/H (ohm/m). The R/H and Xc/H values, transformed into z-scores (ZR and ZXc), were plotted on the graph of the bioimpedance vector using BIVA Software^9^, considering as a reference the healthy Italian population^22^. The position of the vectors of the patients was analyzed in relation to the ellipses of tolerance of 50%, 75%, and 95% of the reference population. Patients were classified as malnourished when the impedance vector was within the lower and upper right quadrants and outside the 75th percentile of the tolerance ellipse along the horizontal axis^23^^,^ 24. To assess the mean value of the impedance vector of the groups, 95% confidence ellipses were used.

### Statistical analysis

The impedance media of the groups were compared using the T² Hotelling test (BIVA 2002 software). Area under the receiver operating curve ROC (AUC) analysis was performed to verify the diagnostic accuracy of BIVA parameters in the identification of patients with malnutrition, based on the 7-point SGA as the reference standard positive and negative likelihood ratios (LR+ and LR− respectively).

The cutoff point performance proposed for the total sample in a previous study^14^ was analyzed, (R/H≥330.05 ohms/m and Z/H≥340.47 ohms/m). The cutoff point for the general sample was chosen because it presented better performance. The area under the curve (AUC) was interpreted as follows: ≥0.90 high accuracy; 0.70-0.90 moderate accuracy; 0.50-0.69 low accuracy; and ≤50 uncertain accuracy^25^. The cutoff points of validity were set as follows: sensitivity and specificity >80%, good validity; sensitivity or specificity <80% but both >50%, fair validity; sensitivity or specificity <50%, poor validity. Two-tailed p-values <0.05 were considered statistically significant^26^. In addition, Cohen's Kappa (κ) was used to assess the agreement between BIVA and the 7-point SGA. The result was interpreted considering Kappa values <0.20 (weak agreement), 0.20 to <0.40 (regular agreement), 0.40 to <0.60 (moderate agreement), 0.60 to <0.80 (good agreement), and 0.80 (almost perfect agreement)^27^. P value <0.05 was defined as statistically significant. All statistical analyses were performed using two-side tests in STATA v.12.0 software.

## Results

We evaluated 104 HD patients; their mean age was 51.7 (±15.1) years, 52% were male, the main etiology of CKD was hypertensive nephrosclerosis (26%), and the most prevalent comorbidity was Systemic Arterial Hypertension (55%). The average KT/v was 1.37 (±0.25). The age and duration of HD in the group with malnutrition were higher than those in the well-nourished group.

Patients with malnutrition had lower serum albumin and creatinine concentrations. The prevalence of malnutrition by BIVA was 25.96% while that by the SGA was 19.23% (Table 1). In addition, 70% of patients ended the HD session hydrated. Among dehydrated patients, 97% were well nourished according to the 7-point SGA. Only one patient had hyperhydration (results not shown in the table).

**Table 1:** Clinical and anthropometric parameters of patients with chronic kidney disease on hemodialysis according to nutritional status by the 7-point subjective global assessment

	Total (n = 104)	Well-nourished (80.77%)	Malnourished 20 (19.23%)	p
Male sex - n (%)	54 (52)^a^	43 (80)	11 (20)	0.762
Age (in years)	51.7 ± 15.1^b^	49.8 ± 14.8	59.6 ± 14.2	**0.009**
HD time (months)	44.5(20.0-89.0)^c^	38.0(18.5-81.5)	65.0(37.0-125.5)	**0.015**
Causes of CKD - n (%)				
Hypertensive nephrosclerosis	26 (25)	21 (25)	5 (25)	0.091
Glomerulonephritis	11 (10)	10 (12)	1 (5)	
Diabetic nephropathy	13 (13)	9 (11)	4 (20)	
Others and undetermined	54 (52)	44 (52)	10 (50)	
Comorbidities - n (%)				
Systemic arterial hypertension	55 (53)	44 (52)	11 (55)	0.262
Diabetes and hypertension	20 (19)	14 (17)	6 (30)	
Diabetes	3 (3)	2 (2)	1 (5)	
Others or without comorbidities	26 (25)	24 (29)	2 (10)	
Urea KT/v	1.37 ± 0.25	1.39 ± 0.23	1.35 ± 0.27	0.628
Biochemical parameters				
Hemoglobin (g/dL)	11.5(10.4-13,0)	10.5(11.5-13.0)	12.0(10.1-12.9)	0.798
Albumin (g/dL)	3.7 ± 0.5	3.7 ± 0.4	3.4 ± 0.6	**0.003**
Creatinine (mg/dL)	11.2 ± 3.1	11.5 ± 3.2	9.2 ± 2.5	**0.044**
Post urea (mg/dL)	29.5(22.0-42.5)	30.0(21.5-42.5)	28.5(22.5-42.5)	0.888
Anthropometric data				
Dry weight (kg)	65.10 ± 13.82	65.57 ± 14.47	63.10 ± 10.75	0.476
IWG (kg)	1.8 ± 0.9	1.8 ± 0.9	1.8 ± 0.7	0.794
BMI (kg/m^2^)	24.3(21.7-27.4)	24.6(21.7-27.4)	24.6(21.7-27.4)	0.400
BIVA classification				
Well nourished	77 (74)	63 (82)	14 (18)	0.647
Malnourished	27 (26)	21 (78)	6(30)	
Dehydrated	31 (30)	30 (97)	1 (3)	**0.021**
Hydrated	73 (70)	54 (74)	19 (26)	

HD: Hemodialysis, CKD: Chronic Kidney Disease, KT/v hemodialysis dose, IWG: Interdialytic Weight Gain, BMI: Body Mass Index. Values expressed in:

a absolute (relative) frequency;

b mean ± standard deviation,

c median (interquartile range), with significant difference for p≤0.05, Chi-Square, Student's t-test for independent samples, or Mann-Whitney test.

BIVA diagnosed malnutrition with a sensitivity of 30.00% using the 7-point SGA as the reference standard. The specificity for identifying the well-nourished was 85.70%. The accuracy (AUC=0.604, CI 95% 0.490-0.726) and agreement (κ=0.21; p=0.016) between the BIVA criteria and 7p-SGA in diagnosing malnutrition were low and regular, respectively. The estimated prevalence of malnutrition based on the combination of the established cutoff points for R/H and Z/H was 70.00% among the total sample. The sensitivity obtained by the analysis using the pre-established cutoff was 72.20% and the specificity was 30.50, the accuracy was low (AUC=0.514, CI 95% 0396-0631), agreement was week (κ=0.013; p=0.410), and the positive predictive value was 18.60 (Table 2).

**Table 2:** Accuracy and concordance of the BIVA criteria with the 7-point subjective global assessment

Criterion	> 75th percentile*	R/H≥330.05 and Z/H≥340.47**
Sensitivity (%)	30.0	72.2
Specificity (%)	85.7	30.5
Positive predictive value (%)	36.8	18.6
Negative predictive value (%)	84.7	83.3
Positive likelihood ratio (LR+)	2.45	1.04
Negative likelihood ratio (LR-)	0.758	0.911
Area under the curve (AUC)	0.604 (95%CI 0.490-0.726)	0.514 (95%CI 0.396-0.631)
Kappa value (p)	0.21(0.016)	0.013 (0.410)

BIVA: Bioelectrical impedance vector analysis. Area under the receiver operating curve (ROC) analysis. Comparison test with a reference standard; categorized variables.

* Distribution of patients on resistance-reactance graph^23^;

** previously established cutoff points^14^.

**Figure 1:**
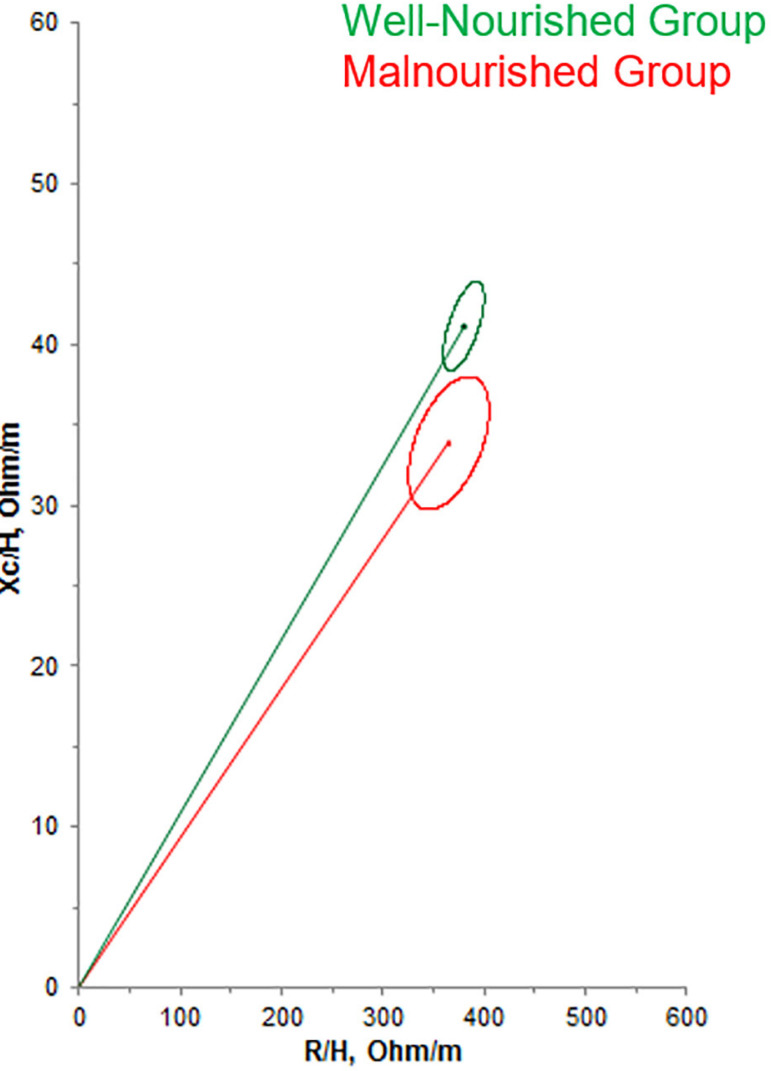
Impedance vectors with 95% confidence ellipses after hemodialysis in the well-nourished (green line) and malnourished (red line) groups according to the 7-point Subjective Global Assessment (p<0.05). Xc/H: height-corrected reactance, R/H: height-corrected resistance.

**Figure 2:**
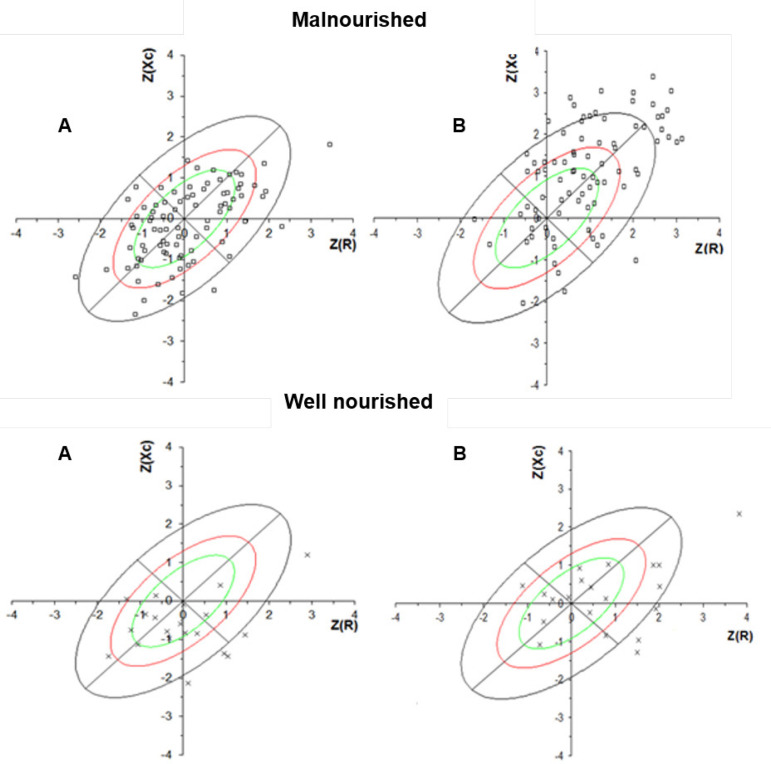
Position of the vector for well-nourished and malnourished patients by 7-point Subjective Global Assessment, before (A) and after (B) hemodialysis. Impedance measures were transformed into Z-scores.

The confidence ellipses representative of the R/H and Xc/H values showed that there was a difference between the vectors of the malnourished and well-nourished groups by the 7-point SGA, since they did not overlap (p<0.05) (Figure 1). In practice, this analysis indicates that well-nourished and malnourished individuals (by the 7-point SGA) have distinct cellularity. In addition, the position of the individual vector before and after the HD session demonstrates that there was a shift parallel to the major axis due to an increase in Z (R) and Z (Xc), which shows a change in hydration status after removal of extracellular fluid (Figure 2).

In the evaluation performed before and after HD, the scores of Xc and R showed a significant increase after HD. In the intergroup analysis, only the Xc variable showed a significant difference, being greater in the well-nourished group both before and after HD (Table 3).

**Table 3:** Sample characteristics before and after the hemodialysis session according to nutritional status defined by the 7-point subjective global assessment

Variáveis	Well nourished			Malnourished			p (Pre HD Intergroup)	p (Post HD Intergroup)
	Pré HD	Pós HD	p	Pré HD	Pós HD	p		
Weight	67.3 ± 14.7	65.6 ± 14.5	**<0.001**	64.8 ± 10.8	63.1 ± 10.7	**<0.001**	0.473	0.476
Z(R)	0.05 ± 1.0	1.0 ± 1.1	**<0.001**	-0.2 ± 1.1	0,7 ± 1,2	**<0,001**	0,854	0,416
Z(Xc)	<0.001	0.854	**0.416**	-0,6 ± 0,7	0,2 ± 0,9	**<0,001**	**0,021**	**0,002**

HD: Hemodialysis, R: Resistance: Xc: Reactance, Z: Z score. Values expressed in: mean ± standard deviation, with significant difference for p≤0.05. Student's t-test for independent samples or Mann-Whitney test.

## Discussion

Our results show low accuracy and week agreement between BIVA and the 7-point SGA results. The BIVA parameters R/H and Z/H demonstrated greater sensitivity than specificity in the identification of malnutrition, but low AUC. These results may have occurred because the 7-point SGA assesses aspects of up to six previous months, whereas BIVA assesses the cellularity and hydration of the present time in the analysis. Furthermore, the vector position of each patient shows the shift of the quadrant before and after the HD session, regarding hydration.

Patients who were malnourished according to the 7-point SGA showed less cellularity by the BIVA. This result was expected since cellularity is evaluated by means of Xc (which offers a measure of opposition to the current flow caused by the capacitance produced by the cell membrane)^28^^,^ 29. Since HD patients are exposed to protein catabolism and lean mass depletion, supported by the wasting concept^30^, malnutrition was expected to be positively correlated with cellularity^12^. This agreement, in clinical practice reinforces the complementarity of BIVA in relation to the 7-point SGA, since the results were concordant. Some patients with low cellularity were not identified by the SGA, reinforcing that those changes on the cellular level may occur before anthropometric and biochemical changes^14^.

The prevalence of malnutrition based on the 7-point SGA (19.23%) in the present study was lower than those reported in the literature for these patients, which ranged from 30 to 50%^3^^,^ 31 ^-^ 33. The main reason for this difference is that the inclusion and exclusion criteria used in the present study contributed to a more homogeneous and clinically stable population. Having an older age was not an exclusion criterion, but the population was quite young.

Two studies in Italian patients compared th BIVA with the three-point and 7-point SGA^13^^,^ 34 and both found a positive association with malnutrition; moreover, the study by Piccoli et al.^13^, proposed the graphical representation of the tolerance ellipses using data of R and Xc from the BIA. This study was carried out with 130 HD patients, and a difference was found based on the graphical representation of the non-overlapping confidence ellipses of the well-nourished and malnourished groups, as observed in the present study. The second study^33^ assessed food intake and prevalence of malnutrition in 52 HD patients. There was also a prospective Brazilian study with a 2-year follow-up^14^ that found agreement of BIVA with the three-point SGA and recommended the use of cutoff points as a reference. In this study, we found an area under the ROC curve smaller than that observed with the BIVA classification. The performance of this cutoff point may not have been as good as in the original study^14^ because of the characteristics of the sample, in which the mean age and percentage of men were higher than in the present study. The BIVA parameters R/H and Z/H showed higher sensitivity than specificity in identifying malnutrition, but these cutoff points showed low accuracy.

As most patients on HD are anuric, fluid management, interdialytic weight gain (IWG) determination and hydration status after dialysis are information that would contribute to the treatment and quality of life of the patient^12^^,^ 28. Water imbalance must be assessed at each session because both volume overload and dehydration are undesirable situations and can bring health risks when they occur chronically, such as in left ventricular hypertrophy and intradialytic hypotension, respectively^12^. In contrast to what is described in the literature^28^, dehydration was more prevalent in the present study, and a small percentage of hyperhydration was found. It is important to note that dehydration is a worrisome a condition as hyperhydration and that both conditions must be recognized early^28^. The 7-point SGA, although it includes a physical examination, is not an accurate method of assessing hydration status^18^. In the present study, 97% of dehydrated patients were classified as well-nourished.

The state of hypervolemia present in the patients before the HD session and confirmed by the IWG did not affect the sensitivity of the BIVA for malnutrition detection in the present study. Confidence ellipses from before and after the HD session show a migration of all patients (well-nourished and malnourished) to the quadrant indicating lower hydration. This difference in position was expected as excess extracellular fluid is removed during the HD session. Therefore, the decrease in total body water (TBW) results in increased resistance. As with Xc, the increase indicates a greater number of cells per tissue unit^13^. A study that analyzed BIVA before and after the HD session also reported a difference in hydration status, expressed in the graph as a change in the patient's quadrant^13^.

A limitation of the study is the use of the 7-point SGA as the only parameter to assess nutritional status and sensitivity of BIVA. Although the 7-point SGA is the recommended parameter^4^, it represents a subjective assessment that affects the accuracy of the method. Comparison with more accurate methods, such as dual energy x-ray absorptiometry (DXA), is suggested for future studies.

This study showed that there are differences in the use of the 7-point SGA and the BIVA to detect malnutrition in CKD patients on HD. Our results show that BIVA parameters R/H and Z/H provide low accuracy in diagnosing malnutrition using the 7-point SGA as reference standard. However, the analysis indicated that well-nourished and malnourished individuals (7-point SGA) had different cellularity according to the BIVA. Therefore, BIVA must be used in conjunction with other methods to diagnose malnutrition, as BIVA can provide cellularity and dehydration data that complement the 7-point SGA and are especially important for monitoring the medical history of the HD patient.

Longitudinal studies using BIVA are needed to monitor the nutritional status of HD patients. We believe that this monitoring will improve the quality of life of patients as BIVA provides cellularity and hydration data which are important for determining dry weight. We recommend BIVA for monitoring cellularity and hydration, two important aspects for the HD population. Therefore, the practical application of BIVA is to monitor nutritional status at the cellular level and hydration. These are two important parameters for medical history, because they prevent the risk of nutritional and cardiovascular complications by revealing changes that cannot be detected in the short term by other methods, such as 7-point SGA.
